# Bone Marrow Suppression by c-Kit Blockade Enhances Tumor Growth of Colorectal Metastases through the Action of Stromal Cell-Derived Factor-1

**DOI:** 10.1155/2012/196957

**Published:** 2011-10-02

**Authors:** Kathrin Rupertus, Gudrun C. Y. Haberl, Claudia Scheuer, Michael D. Menger, Martin K. Schilling, Otto Kollmar

**Affiliations:** ^1^Department of General, Visceral, Vascular and Pediatric Surgery, University of Saarland, 66421 Homburg, Germany; ^2^Department of Hematology, Oncology and Immunology, University of Tübingen, 72074 Tübingen, Germany; ^3^Institute for Clinical and Experimental Surgery, University of Saarland, 66421 Homburg, Germany

## Abstract

*Background*. Mobilization of c-Kit^+^ hematopoietic cells (HCs) contributes to tumor vascularization. Whereas survival and proliferation of HCs are regulated by binding of the stem cell factor to its receptor c-Kit, migration of HCs is directed by stromal cell-derived factor (SDF)-1. Therefore, targeting migration of HCs provides a promising new strategy of anti-tumor therapy. *Methods*. BALB/c mice (*n* = 16) were pretreated with an anti-c-Kit antibody followed by implantation of CT26.WT-GFP colorectal cancer cells into dorsal skinfold chambers. Animals (*n* = 8) additionally received a neutralizing anti-SDF-1 antibody. Animals (*n* = 8) treated with a control antibody served as controls. Investigations were performed using intravital fluorescence microscopy, immunohistochemistry, flow cytometry and western blot analysis. *Results*. Blockade of c-Kit significantly enhanced tumor cell engraftment compared to controls due to stimulation of tumor cell proliferation and invasion without markedly affecting tumor vascularization. C-Kit blockade significantly increased VEGF and CXCR4 expression within the growing tumors. Neutralization of SDF-1 completely antagonized this anti-c-Kit-associated tumor growth by suppression of tumor neovascularization, inhibition of tumor cell proliferation and reduction of muscular infiltration. *Conclusion*. Our study indicates that bone marrow suppression via anti-c-Kit pretreatment enhances tumor cell engraftment of colorectal metastases due to interaction with the SDF-1/CXCR4 pathway which is involved in HC-mediated tumor angiogenesis.

## 1. Introduction

Angiogenesis is one of the crucial steps in tumor progression and metastasis [1, 2]. Due to the lack of oxygen supply and the accumulation of toxic products, avascular tumors and tumor metastases cannot grow beyond a critical size of *∼*1-2 mm and thus will stay clinically occult [1, 3, 4]. Therefore, only a small proportion of circulating cancer cells finally forms macroscopic tumors [5]. Tumor vessels can grow by sprouting of preexisting host vessels, intussusception or incorporation of bone marrow-derived endothelial progenitor cells (EPCs) which are a subset of hematopoietic cells (HCs). This mechanism is called vasculogenesis and mimics embryonic angio-development [2].

Although EPCs incorporate into tumors in only small numbers, targeting EPCs has been shown to be effective in reducing tumor angiogenesis and tumor growth in experimental models [6]. One of the most important factors mediating survival and proliferation of HCs is the stem cell factor (SCF) which binds to the c-Kit receptor on the surface of HCs. Okamoto et al. have shown that bone marrow suppression by anti-c-Kit treatment induces a delay in tumor angiogenesis due to the inhibition of angiogenic sprouting in colon tumors and that c-Kit blockade suppresses tumor growth of subcutaneously implanted prostate carcinomas (PC3) by inhibition of tumor angiogenesis regulated by HCs [7].

Recruitment of HCs and EPCs to avascular areas is orchestrated by different angiogenic growth factors and cytokines [8], predominantly by the CXC-chemokine stromal cell-derived factor (SDF)-1 and its receptor CXCR4 [9, 10]. HCs and EPCs fluctuate to peripheral organs in antiphase with the expression of SDF-1 within the bone marrow microenvironment [11] and migrate alongside a chemotactic gradient towards higher concentrations of SDF-1, for example, sites of injury and tumor tissue [12–14]. Furthermore, it has been demonstrated that SDF-1-mediated recruitment of c-Kit-positive cells to the periphery is dependent on cofactors, including tumor necrosis factor-*α* and the endothelial nitric oxide synthase (eNOS) [12].

However, the impact of circulating HCs and EPCs on vasculogenesis and sprouting angiogenesis in tumor angio-development is still controversially discussed in the literature [15–17], and the role of the SDF-1/CXCR4 pathway is not yet fully understood. Therefore, in the presented study we analyzed the influence of bone marrow suppression by anti-c-Kit treatment combined with SDF-1 neutralization on tumor cell engraftment and neovascularization using a murine model of colorectal tumor metastasis.

## 2. Materials and Methods

### 2.1. Tumor Cell Line and Culture Conditions

The CT26 cell line is an N-nitroso-N-methyl-urethane-induced undifferentiated adenocarcinoma of the colon, syngeneic with the BALB/c mouse. For our studies [18], the CT26.WT cells (ATCC CRL-2638, LGC Promochem GmbH, Wesel, Germany) were transfected with the enhanced GFP expression vector pEGFP-N1 (Clontech) with the use of CLONfectin (Clontech, Palo Alto, CA, USA) according to the manufacturer's instructions. For the individual experiments, CT26.WT-GFP cells were grown in cell culture as monolayers in RPMI-1640 medium with 2 mM L-glutamine (Sigma Aldrich Chemie GmbH, Taufkirchen, Germany) supplemented with 10% fetal calf serum (FCS Gold, PAA Laboratories GmbH, Cölbe, Germany), 100 U/mL penicillin, and 100 *μ*g/mL streptomycin (PAA Laboratories GmbH). The cells were incubated at 37°C in a humidified atmosphere containing 5% CO_2_, and only cells of the first three serial passages after cryostorage were used. At the day of implantation, tumor cells were harvested from subconfluent cultures (70 to 85%) by trypsinization (0.05% Trypsin and 0.02% EDTA, PAA Laboratories GmbH) and washed twice in phosphate-buffered saline solution (PBS).

### 2.2. Animals

Experiments were performed after approval by the local governmental ethic committee and conformed to the United Kingdom Coordinating Committee on Cancer Research (UKCCCR) Guidelines for the Welfare of Animals in Experimental Neoplasia (as described in 1998 in *Br J Cancer *77: 1–10) and the Guide for Care and Use of Laboratory Animals (Institute of Laboratory Animal Resources, National Research Council; NIH Guide, Vol. 25, No. 28, 1996). Female BALB/c mice (Charles River Laboratories GmbH; Sulzfeld, Germany) with a body weight (BW) of 18–20 g were used. The animals were housed in single cages at room temperature of 22–24°C and at a relative humidity of 60–65% with a 12-hour light/dark cycle environment. The mice were allowed free access to drinking water and standard laboratory chow (Altromin; Lage, Germany).

### 2.3. Experimental Model—Dorsal Skinfold Chamber

To allow repetitive analyses of the microcirculation of the growing tumors, the dorsal skinfold chamber model was used for intravital microscopy as described previously in detail [19]. For operative procedures, animals were anesthetized by intraperitoneal injection of 90 mg/kg BW ketamine (Ketavet, Parke Davis; Freiburg, Germany) and 20 mg/kg BW xylazine (Rompun, Bayer; Leverkusen, Germany). The chamber, consisting of two symmetrical titanium frames, was positioned to sandwich the extended double layer of the dorsal skin. One layer of skin and subcutis was completely removed in a circular area of 15 mm diameter. The remaining layers, consisting of the epidermis, subcutaneous tissue, and striated skin muscle, were covered with a glass coverslip incorporated into one of the titanium frames. The animals tolerated the chambers well and showed no signs of discomfort or changes in sleeping and feeding habits. After a 48-hour recovery period, the animals were reanesthetized. For tumor cell implantation, the coverslip of the chamber was temporarily removed and 1 × 10^5^ CT26.WT-GFP cells were implanted onto the surface of the striated muscle tissue within the chamber. Immediately after cell implantation, the chamber tissue was covered again with the coverslip [20–22].

### 2.4. Experimental Protocol

Animals were assigned to three different groups: the first group (cKit-Ab; *n* = 8) received a pretreatment with a monoclonal anti-c-Kit receptor antibody (ACK45, BD Biosciences, Heidelberg, Germany) by daily intraperitoneal injections starting 4 days before tumor cell implantation. The ACK45-antibody was given four times at a dose of 1 mg/kg BW daily as described by Okamoto et al. [7]. Animals of the second group (cKit/SDF1-Ab; *n* = 8) were also pretreated with ACK45 as described above. Additionally, these animals received 1 mg/kg BW of a monoclonal mouse anti-mouse SDF-1 antibody (MAB310, R and D Systems, Wiesbaden, Germany). MAB310 application was performed intraperitoneally, starting at the day of tumor cell implantation (d0) and repeated every second day thereafter until day 12. The third group (control; *n* = 8) served as control and received pre- and posttreatment the same amount of the corresponding isotype-matched IgG control antibodies (A95-1, BD Biosciences, and MAB002 R&D Systems). All animals underwent repetitive intravital microscopic analyses directly (d0) as well as 5, 8, 11, and 14 days after tumor cell implantation. At the end of the experiment (day 14), the chamber with the tumor tissue was harvested for histology and immunohistochemistry.

### 2.5. Intravital Fluorescence Microscopy

Intravital fluorescence microscopy was performed in epi-illumination technique using a modified Zeiss Axio-Tech microscope (Zeiss, Oberkochen, Germany) with a 100-W HBO mercury lamp. Microscopic images were monitored by a charge-coupled device video camera (FK 6990, COHU, Prospective Measurements Inc., San Diego, CA, USA) and were transferred to a video system (VO-5800 PS, Sony, München, Germany) for subsequent off-line analysis. Tumor size, growth kinetics, migration of tumor cell, and neovascularization were analyzed using blue light epi-illumination (450 to 490 nm excitation wavelength and >520 nm emission wavelengths) [20–22].

### 2.6. Microcirculation Analysis

Microcirculatory parameters were assessed off line by frame-to-frame analysis of the videotaped images using a computer-assisted image analysis system (CapImage, Zeintl Software, Heidelberg, Germany). The fluorescent labeling of the tumor cells allowed precise delineation of the tumor from the surrounding host tissue. It also enabled for distinct identification of individual tumor cells to study tumor cell migration. At each observation time point, the surface of the fluorescently labeled tumor mass within the chamber was scanned for determination of the tumor size (given as tumor area in mm^2^). Eight regions of interest (ROIs) were randomly chosen next to the tumor margin. The number of migrating cells was counted, and the distance to the tumor margin was measured (given in *μ*m).

Microcirculation was quantified as described before in detail [22]. First, eight representative ROIs within the tumor margin were chosen and analyzed for microcirculation parameters. In these ROIs, the onset of angiogenesis, that is, the existence of angiogenic buds, sprouts, and newly formed blood vessels, was documented and scored 0 to 8, with 0 indicating existence of newly formed tumor microvessels in none of the ROIs and 8 indicating their existence in all of the ROIs. Functional capillary density (given in cm/cm²) of the tumor microvessels was measured to quantify the angiogenic activity. This parameter was defined as the length of red blood cell perfused microvessels per observation area and was analyzed within the eight ROIs of the tumor margin and within four additional ROIs of the tumor center. Diameters of the newly formed tumor microvessels were measured perpendicularly to the vessel path (given in *μ*m). To study vascular permeability of the newly formed tumor microvessels, petechial bleedings were documented in each of the ROIs, and given as percentage of all the ROIs analyzed [20, 21, 23].

### 2.7. Histology and Immunohistochemistry

At the end of the experiment (day 14), the tumor and the adjacent host tissue were harvested and immediately fixed in formalin. For light microscopy, formalin-fixed biopsies were embedded in paraffin. Sections of 5 *μ*m were cut and stained with hematoxylin and eosin (HE) according to standard procedures to analyze tumor growth characteristics. Tumor cell invasion of the muscular layer on the surface of the dorsal skinfold chamber was quantified over the entire tumor basis and given as percentage of the length of the tumor basis.

To study tumor cell proliferation and apoptotic cell death, proliferating cell nuclear antigen (PCNA) and cleaved caspase-3 were stained using indirect immunoperoxidase techniques. Therefore, deparaffinized sections were incubated with 3% H_2_O_2_ and 2% goat normal serum to block endogenous peroxidases and unspecific binding sites. A monoclonal mouse anti-pan PCNA antibody (PC10, DakoCytomation, Hamburg, Germany) and a polyclonal rabbit anti-mouse cleaved caspase-3 antibody (Asp175, Cell Signaling Technology, Frankfurt, Germany) were used as primary antibodies. Goat anti-mouse and goat anti-rabbit POD-conjugated antibodies were used as secondary antibodies for streptavidin-biotin-complex peroxidase staining. 3,3′ diaminobenzidine (DakoCytomation) served as chromogen. Sections were counterstained with Hemalaun according to Mayer and examined by light microscopy.

To assess the expression of CD31 as a marker for endothelial cells, zinc fixative fixed paraffin sections of tumor tissue were used. After incubation with 3% H_2_O_2_ and 2% goat normal serum to block endogenous peroxidases and unspecific binding sites, deparaffinized sections were incubated with a rat anti-mouse CD31 antibody (MEC 13.3, BD Biosciences). A polyclonal goat anti-rat IgG antibody (BD Biosciences) was used as secondary antibody. Colorimetric detection was performed using 3,3′ diaminobenzidine (DakoCytomation) substrates. Sections were counterstained with Hemalaun according to Mayer and examined by light microscopy.

### 2.8. Flow Cytometric Analysis of CT26.WT-GFP Cells

FACScan (Becton Dickinson, Mountain View, CA, USA) analysis was performed to assess the expression of c-Kit on the CT26.WT-GFP cells in triplicate. Cells were fixed with 2% formalin, washed twice with PBS, resuspended in FACS buffer and incubated with an FITC-conjugated rat anti-mouse IgG2b c-Kit antibody (1 : 50; 553354, BD Biosciences) or an FITC-conjugated isotype-matched control antibody (553988, BD Biosciences). Cells were washed again and then maintained in 2% paraformaldehyde in PBS. Tumor cells were selectively analyzed for their fluorescence properties using the CellQuest data handling program (BD Biosciences) with assessment of 5000 events per sample. The flow cytometer was calibrated with fluorescent standard microbeads (CaliBRITE Beads, BD Biosciences).

### 2.9. Western Blot Analysis

To study protein expression patterns, additional Western blot analyses were performed on tumor specimen gained from the dorsal skinfold chamber. Therefore, 12 additional animals received CT26.WT-GFP tumor cell implantation in the dorsal skinfold chamber and were assigned to the three groups as described above. Eight animals were pretreated with the anti-c-Kit antibody (ACK45, BD Biosciences). Four of these pretreated animals received additional treatment with the neutralizing anti-SDF-1 antibody (MAB310, R and D Systems) starting at the day of tumor cell implantation. Four animals received the same amount of the corresponding isotype-matched control antibody (A95-1, BD Biosciences).

For whole protein extracts and Western blot analysis of the expression of vascular endothelial growth factor (VEGF), the chemokine receptor CXCR4 and eNOS, CT26.WT-GFP-tumors were completely removed from the skinfold at day 5. The tumor tissue samples were homogenized separately in lysis buffer and a protease inhibitor cocktail (Sigma, Taufkirchen, Germany), incubated on ice and centrifuged at 16.000 ×g. The supernatant was saved as whole protein extract fraction. Protein concentrations were determined using the Lowry assay with bovine serum albumin as standard. Ten microgram protein per lane were separated discontinuously on sodium dodecyl sulfate polyacrylamide gels (10% SDS-PAGE) and transferred to a polyvinyldifluoride membrane (0.2 *μ*m, BioRad, München, Germany). After blockade of nonspecific binding sites, membranes were incubated with an anti-VEGF antibody (A-20, Santa Cruz, Heidelberg, Germany), an anti-CXCR4 antibody (ab2074, Abcam, Heidelberg, Germany), or an anti-eNOS antibody (BD Transduction Lab., Heidelberg, Germany) followed by the corresponding secondary peroxidase-conjugated antibodies (GE Healthcare, Freiburg, Germany and Santa Cruz). Protein expression was visualized by means of luminol enhanced chemiluminescence (ECL, GE Healthcare) and exposure of the membranes to a blue-light-sensitive autoradiography film (Hyperfilm ECL, GE Healthcare). Signals were densitometrically assessed (BioRad, Gel-Dokumentationssystem) and normalized to *β*-actin signals (mouse monoclonal anti-*β*-actin, Sigma) to correct for unequal loading.

### 2.10. Statistical Analysis

All values are expressed as means ± SEM. After proving the assumption of normality and homogeneity of variance across groups, differences between groups were calculated by a one-way analysis of variance (ANOVA) followed by the appropriate post hoc comparison, including correction of the alpha error according to Bonferroni probabilities to compensate for multiple comparisons. Overall statistical significance was set at *P* < 0.05. Statistical analysis was performed with the use of the software package SigmaStat (SPSS Inc, Chicago, Ill).

## 3. Results

### 3.1. Tumor Growth

**Figure 1:**
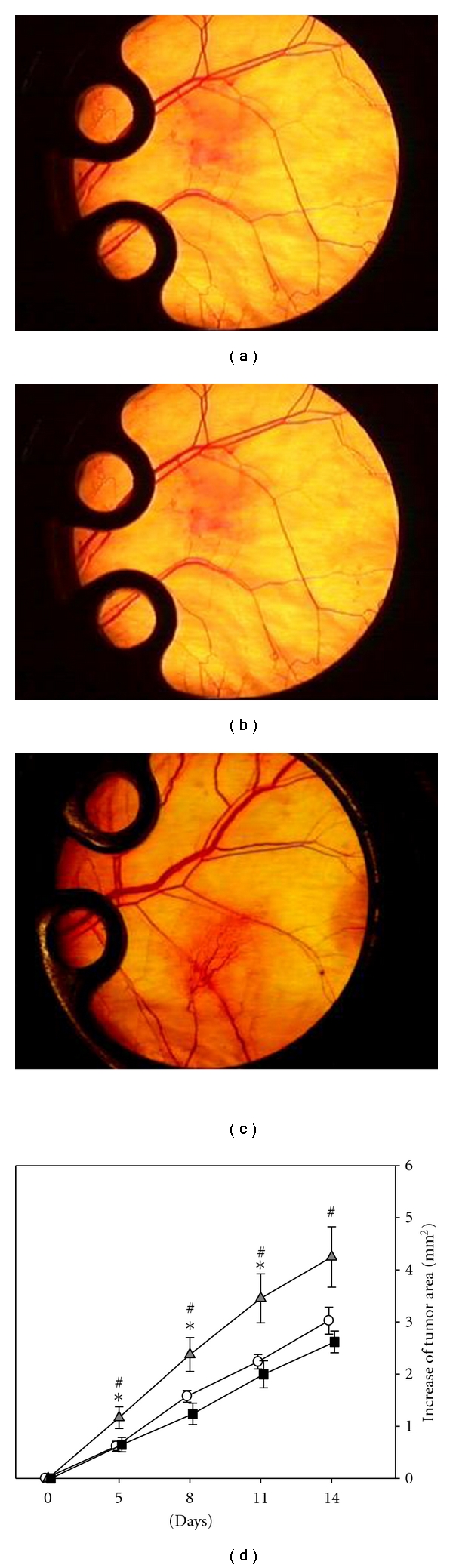
Time course of tumor growth of CT26.WT-GFP tumors in the dorsal skinfold chamber. Stereomicroscopy photographs of representative 14 days old tumors after either treatment with an isotype-matched control antibody (a), pretreatment with anti-c-Kit (b) alone, or pretreatment with anti-c-Kit followed by anti-SDF-1 treatment (c). Quantitative analysis of the tumor area (d) displayed progressive tumor growth in all groups. After anti-c-Kit pretreatment (grey triangles), tumor growth was significantly accelerated compared to controls (white circles). Of interest, additional neutralization of SDF-1 (black squares) completely blunted this anti-c-Kit-associated enhancement of tumor growth. Mean ± SEM; *P < 0.05 versus control; ^#^ *P* < 0.05 versus anti-c-Kit. Original magnification (a)–(c) ×4.

All animals had an uneventful postoperative recovery and tolerated well the dorsal skinfold chamber implantation and the repetitive intravital microscopic analyses. The general conditions of the mice were not affected and no changes in feeding or sleeping habits were observed. The take rate of the CT26.WT-GFP cells within the dorsal skinfold chamber was 100%, and progressive tumor growth was observed in all groups throughout the entire observation period (Figures 1(a)–1(c)). Quantitative analysis of the increase of the tumor area revealed a significantly enhanced engraftment of tumor cells and a significant stimulation of tumor growth after pretreatment with the anti-c-Kit antibody compared to controls (*P* < 0.05). Interestingly, additional blockage of SDF-1 completely blunted this enhancement of tumor growth leading to similar tumor sizes as measured in controls (*P* < 0.05; Figure 1(d)).

### 3.2. Angiogenesis and Neovascularization

**Figure 2:**
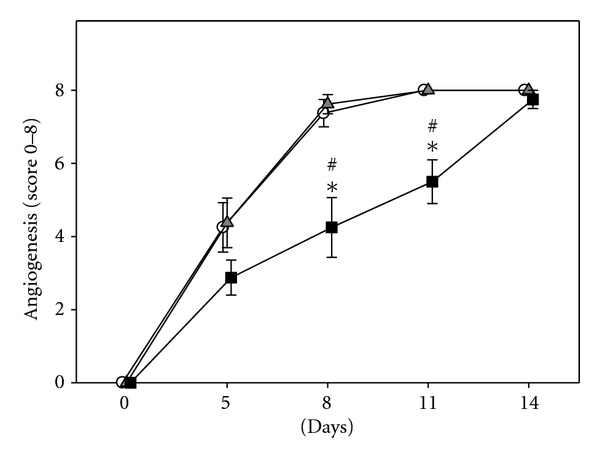
Semiquantitative analysis of the onset of angiogenesis within the CT 26.WT-GFP tumors. Animals were pretreated with anti-c-Kit (grey triangles) alone or anti-c-Kit and anti-SDF-1 neutralizing antibodies (black squares). Animals treated with isotype-matched control antibodies served as controls (white circles). In controls and anti-c-Kit pretreated animals, *∼*50% of the ROIs showed newly developed microvessels 5 days after tumor cell implantation. By day 8, almost 100% of the ROIs were vascularized. Treatment with anti-c-Kit and anti-SDF-1 resulted in a significant delay of angiogenesis with only *∼*50 and *∼*70% of the ROIs showing newly developed microvessels by days 8 and 11. Within these tumors, angiogenesis could be observed in all ROIs only at day 14. Mean ± SEM; *P < 0.05 versus control; ^#^ *P* < 0.05 versus anti-c-Kit.

In control animals, newly developed microvessels could be detected in *∼*50% of the ROIs by day 5 after tumor cell implantation. At day 8, angiogenesis was seen in almost 100% of the ROIs. Pretreatment with the anti-c-Kit antibody did not influence the onset of the angiogenic switch compared to controls. Of interest, blockade of SDF-1 after pretreatment with anti-c-Kit significantly delayed the angiogenic switch, with only *∼*50% of the ROIs showing newly formed microvessels by day 8 and *∼*70% by day 11 (*P* < 0.05). In this group, angiogenesis was observed in all ROIs only at the end of the observation period (Figure 2).

**Figure 3:**
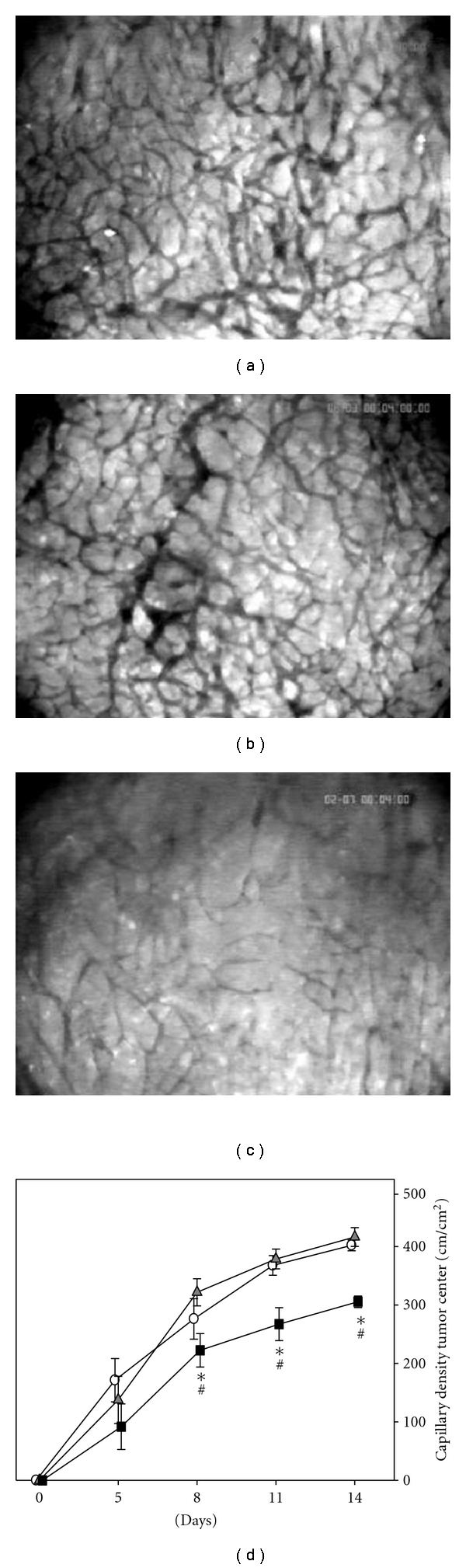
Time course of the functional capillary density within CT-26.WT-GFP tumors in dorsal skinfold chambers analyzed by intravital fluorescence microscopy. Representative fluorescence microscopic images show the network of chaotically arranged microvessels within the tumor center of control animals (a), animals pretreated with anti-c-Kit (b), and animals additionally treated with anti-SDF-1 (c) at day 11 after tumor cell implantation. Quantitative analysis of the functional capillary density (d) revealed a significant inhibition of tumor neovascularization within the tumor center after combined anti-c-Kit and anti-SDF-1 treatment (black squares) compared to controls (white circles). Anti-c-Kit pretreatment alone (grey triangles) did not significantly influence the extent of neovascularization. Mean ± SEM; *P < 0.05 versus control; ^#^ *P* < 0.05 versus anti-c-Kit. Original magnification (a)–(c) ×40.

The differential effects of c-Kit blockade with or without additional SDF-1 blockade were also reflected by quantitative analysis of the microvascular densities of the newly formed tumor vessels. As characteristic for tumor vessels, the vascular network of the tumors consisted of irregularly shaped and chaotically arranged microvessels (Figures 3(a)–3(c)). Whereas no differences of microvascular density within the tumor vasculature could be observed between controls and anti-c-Kit pretreated animals, additional treatment with the anti-SDF-1 antibody significantly decreased the microvascular density within the tumor center compared to the other groups from day 8 until the end of the observation period (Figure 3(d)). Quantitative analysis of the functional microvascular density within the tumor margin showed similar results (data not shown). The functional microvascular density within the tumor margin and the tumor center was not significantly different within the individual treatment groups.

In contrast to the highly vascularized CT26.WT-GFP tumors within the intravital fluorescence microscopy, immunohistological staining for CD31 as a marker for endothelial cells displayed positive staining of only a few cells within tumor microvessels without significant differences between the three groups (data not shown).

As neovascularization is usually associated with vasodilation due to the action of VEGF, microvessel diameters within the tumor were analyzed. However, no overall differences between the diameters of the microvessels within the tumor margin and the tumor center could be observed (Table 1). During the 14 days observation period, diameters slightly increased in all groups until day 11, particularly in the control and anti-c-Kit-pretreated groups. Of interest, at day 14, microvessel diameters within tumors of mice treated with anti-c-Kit and anti-SDF-1 were markedly smaller compared to the other two groups (Table 1).

Petechial bleedings within the areas of angiogenesis of growing tumors are a characteristic indicator of the VEGF action on vascular permeability. To study this VEGF-induced increase of vascular permeability, we analyzed the petechial bleedings within the ROIs by intravital fluorescence microscopy. Microbleedings were observed in all tumors. At day 5, signs of petechial bleedings were observed in *∼*6% of the tumor area of control tumors, whereas petechial bleedings occurred in up to 30% of the tumor area in animals pretreated with anti-c-Kit at that time. In all groups, petechial bleedings were most pronounced 8 days after tumor cell implantation (control: 15–20%, anti-c-Kit: *∼*30%, anti-c-Kit/anti-SDF-1: 35–40%). Comparing the three groups, no statistical significance could be found due to the high standard deviation within the individual groups (data not shown). 

### 3.3. Tumor Cell Migration

As SDF-1 has been demonstrated to exert chemotactic effects on CT26.WT-GFP tumor cells, we additionally focused on migrating tumor cells next to the tumor margin. The migrating tumor cells were easily detectable due to their GFP labeling (Table 2). Starting at day 5 after tumor cell implantation, tumor cell migration was detectable in all tumors until the end of the observation period with slightly increasing distances from the tumor margin. The number of migrating tumor cells ranged between 20 and 25 per representative field during the whole observation period. Treatment with anti-c-Kit alone or additional blockade of SDF-1 did not impair tumor cell migration compared to controls (Table 2).

### 3.4. Tumor Cell Morphology, Proliferation, and Apoptotic Cell Death

**Figure 4:**
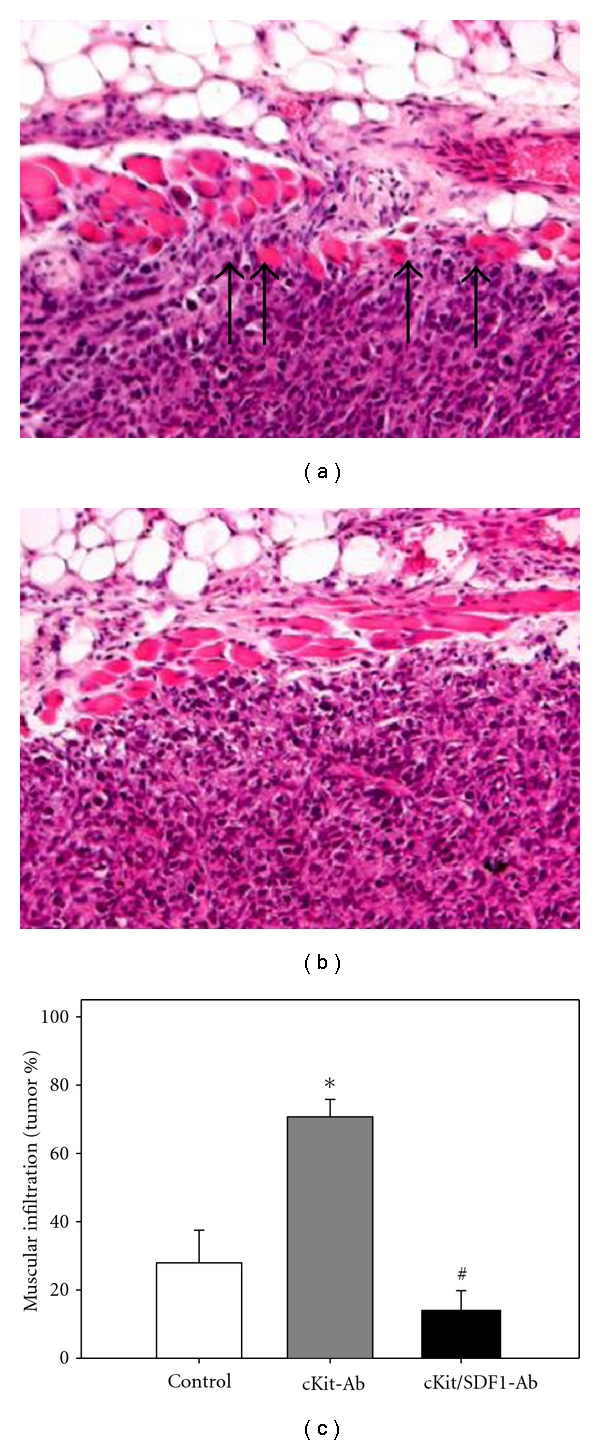
Hematoxylin-eosin staining of CT26.WT-GFP tumors shows solid tumor growth 14 days after tumor cell implantation within the dorsal skinfold chamber. Sections display tumor infiltration through the underlying muscular layer (marked by arrows) after pretreatment with anti-c-Kit (a) and lack of muscular infiltration after pretreatment with anti-c-Kit and additional neutralization of SDF-1 (b). Quantitative analysis of tumor cell invasion of the muscular layer is given as percentage of the length of the tumor. Tumors of animals pretreated with anti-c-Kit showed a significantly pronounced infiltration of the muscular layer compared to controls. Of interest, blockade of SDF-1 after anti-c-Kit pretreatment abrogated this anti-c-Kit-associated increase of tumor cell infiltration. Mean ± SEM; *P < 0.05 versus control; ^#^ *P* < 0.05 versus anti-c-Kit. Original magnification (a, b) ×88.

Histological examinations on hematoxylin-eosin stained sections revealed solid tumor growth within the dorsal skinfold chamber. Signs of malignant tumor growth such as invasion of the adjacent host tissue by the tumor cells were detectable in each group (Figure 4). Quantitative analysis of tumor cell invasion showed a significant increase of tumor cell infiltration through the muscular layer of the dorsal skinfold chamber in tumors of animals which were pretreated with anti-c-Kit compared to controls (*P* < 0.05). Of interest, tumors of animals additionally treated with anti-SDF-1 showed a significant reduction of muscular infiltration compared to anti-c-Kit pretreated tumors (*P* < 0.05), resulting in invasive growth characteristics which were comparable to controls (Figure 4(c)).

**Figure 5:**
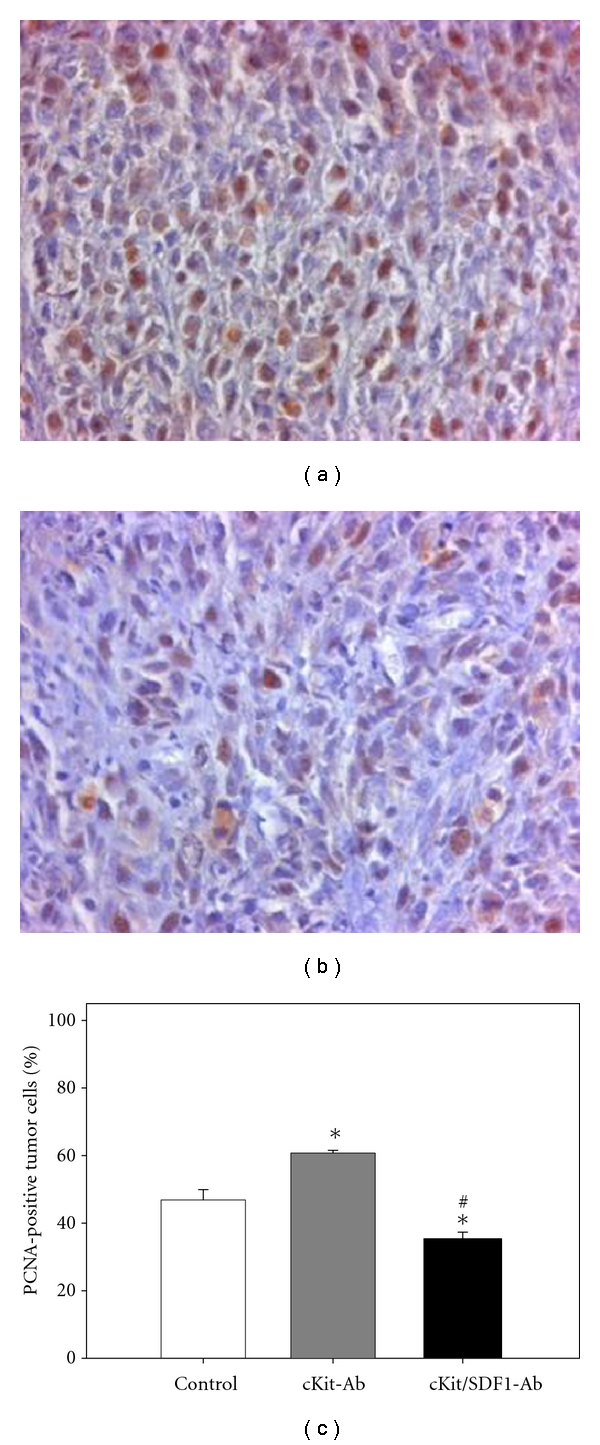
PCNA immunohistochemistry in CT26.WT-GFP tumors at day 14 after tumor cell implantation. Animals were pretreated with anti-c-Kit (cKit-Ab, (a)) alone or anti-c-Kit and anti-SDF-1 neutralizing antibodies (cKit/SDF1-Ab, (b)). Animals treated with an isotype-matched control antibody served as controls (control). Quantitative analysis demonstrated that anti-c-Kit pretreatment significantly increased the rate of proliferating tumor cells compared to controls (c). Of interest, additional neutralization of SDF-1 significantly decreased the amount of proliferating tumor cells compared to controls and anti-c-Kit-treated animals. Mean ± SEM; *P < 0.05 versus control; ^#^ *P* < 0.05 versus anti-c-Kit. Original magnification (a, b) ×175.

PCNA as an indicator of cell proliferation displayed positive staining in 46.9 ± 3.1% of the tumor cells in control animals (Figure 5). After pretreatment with anti-c-Kit, the number of PCNA-positive tumor cells was significantly higher compared to controls (*P* < 0.05, Figures 5(a) and 5(c)). In contrast, additional blockade of SDF-1 resulted in a significantly lower number of PCNA-positive tumor cells compared to controls and to anti-c-Kit pretreated animals (*P* < 0.05, Figures 5(b) and 5(c)).

To study apoptotic cell death, immunohistochemical staining of cleaved caspase-3 products within the tumors was performed. Of interest, 14 days after tumor cell implantation, only a minor fraction of the total number of 351.3 ± 5.2 tumor cells within the high power fields (HPF) showed positive staining for caspase-3. Whereas 0.73 ± 0.13 tumor cells/HPF were positive for caspase-3 in controls, pretreatment with anti-c-Kit reduced apoptotic cell death within the tumors (0.26 ± 0.06 tumor cells/HPF). In tumors of animals additionally treated with anti-SDF-1 antibodies, this effect was even more pronounced (0.15 ± 0.03 tumor cells/HPF, *P* < 0.05).

### 3.5. C-Kit Expression and Western Blot Analysis

FACScan analysis demonstrated that 8.2 ± 0.8% of the CT26-GFP cells transfected with the enhanced GFP expression vector pEGFP-N1 was c-Kit receptor positive.

**Figure 6:**
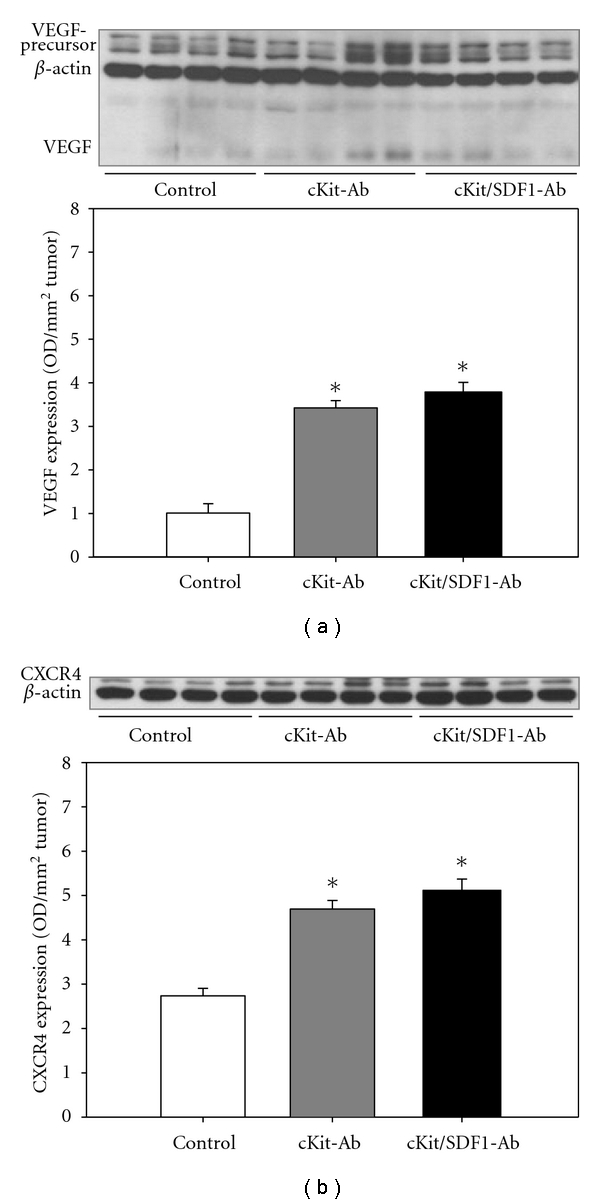
Western blot analysis of VEGF (a) and CXCR4 (b) expression within CT 26.WT tumors five days after tumor cell implantation. Animals were pretreated with anti-c-Kit (cKit-Ab) alone or additionally treated with a SDF-1 neutralizing antibody (cKit/SDF1-Ab). Animals treated with isotype-matched control antibodies served as controls (control). Quantitative analysis showed that VEGF expression was significantly higher after anti-c-Kit pretreatment than in control animals (a). Additional neutralization of SDF-1 had no further effect on the increased VEGF expression. CXCR4 was also significantly higher expressed after anti-c-Kit pretreatment compared to controls and was not further influenced by additional SDF-1 neutralization (b). Mean ± SEM; *P < 0.05 versus control.

At day 5 after tumor cell implantation, the tumors of animals pretreated with anti-c-Kit antibodies showed a significantly higher expression of VEGF and CXCR4 compared to controls (*P* < 0.05; Figures 6(a) and 6(b)). Additional SDF-1 blockade did not further increase VEGF and CXCR4 expression. Furthermore, eNOS expression within the tumors was decreased by anti-c-Kit pretreatment with and without additional anti-SDF-1 treatment compared to controls (data not shown).

## 4. Discussion

The major finding of the present study is that bone marrow suppression by anti-c-Kit treatment significantly enhances tumor cell engraftment of colorectal tumors due to an increase of tumor cell proliferation and invasion. Additional anti-SDF-1 treatment neutralizes this increased tumor outgrowth by inhibition of tumor cell proliferation and tumor neovascularization. These findings suggest that the enhanced tumor growth under the conditions of bone marrow suppression induced by anti-c-Kit treatment is related to the SDF-1/CXCR4 pathway.

Bone-marrow-derived hematopoetic cells (HCs) contribute to physiological and pathological vessel formation. During tumor growth, the release of cytokines and chemokines mediates the recruitment of EPCs and HCs contributing to the early initiation and stabilization of newly formed blood vessels, so-called vasculogenesis [24–26]. However, their precise role has not been fully elucidated yet. HCs and EPCs express stem cell markers such as c-Kit. Whereas many studies indicate that bone-marrow-derived EPCs incorporate into tumor neovessels [25, 27], HCs may promote vasculogenesis via paracrine release of angiogenic factors enhancing the recruitment and incorporation of EPCs into neovessels [28].

In the early phases of tumor growth, 50–90% of the neovessels within the tumor mass are derived from the bone marrow dependent on the tumor type [25]. However, recent reports have already doubted the impact of vasculogenesis from bone-marrow-derived cells for tumor neovascularization and claim an exclusive role for sprouting angiogenesis in tumor blood vessel development [15, 16]. In a model of syngeneic bone marrow transplantation, Patil et al. demonstrated that GPF-expressing c-Kit^+^ bone-marrow-derived progenitor cells are recruited to subcutaneously implanted Lewis lung carcinoma but do not directly contribute to microvascular structure. They, therefore, concluded that even if circulating HCs and EPCs home to sites of tumor growth, they do not contribute to tumor angiogenesis [29]. In a model of subcutaneously implanted prostate carcinoma, Okamoto et al. observed an accumulation of HCs around newly formed blood vessels, but did not find these cells to be part of the tumor microvessels themselves. Because of these findings, the authors postulated a stabilizing and supportive role of HCs for the developing vascular network [7].

In the present study, intravital fluorescence microscopy showed highly vascularized CT26.WT-GFP tumors 14 days after tumor cell implantation. However, immunohistological staining using the endothelial cell marker CD31 displayed positive staining of only a few endothelial cells within these tumors. This finding reflects the diverging structure between normal microvessels and tumor neovessels which consist of only cancer cells or a mosaic of cancer and endothelial cells [2].

Previous experimental studies in mice have shown that a depletion of the myeloid and erythroid cell lineages including EPCs and HCs from the bone marrow could be performed by daily injection of 1 mg/kg ACK2, an antagonistic anti-c-Kit antibody, which blocks the function of c-Kit without cytotoxic side effects on c-Kit^+^ cells [30, 31]. Of interest, whereas no polymorphonuclear cells or erythroblasts were present in the bone marrow of these ACK2-treated animals, B lineage cells continued to grow to fill the space from which myeloid and erythroid progenitor cells were purged [30, 31]. Moreover, the reduction rate of the colony forming cells within the bone marrow was dependent on the amount of anti-c-Kit antibodies [30]. In the present study, we, therefore, used the anti-c-Kit antibody in a dosage of 1 mg/kg BW for the induction of bone marrow suppression over a time period of 4 days to avoid surgical complications and death of the animals resulting from the immune incompetence by bone marrow depletion.

As shown by Okamoto et al., bone marrow suppression by anti-c-Kit pretreatment over a time period of 4 days before subcutaneous implantation of colon tumor cells induced leucopenia which was still detectable 10 days after the last injection [7]. Although tumor growth and sprouting of tumor vessels were slightly reduced in these studies of Okamoto et al. during the first 5–7 days, tumor growth was rapidly reinitiated as the number of circulating HCs in the peripheral blood increased again [7]. Therefore, the authors concluded that HCs migrating into the tumor mass promote the initiation of tumor neovascularization. In our present study, although neoangiogenesis was not influenced after administration of anti-c-Kit, tumor growth was stimulated as a result of an increased tumor cell proliferation and invasion. This observation might be the result of immunological mechanisms that suppress tumor growth in animals with an intact immune system. C-Kit blockade for the induction of bone marrow suppression concurrently stimulates B lymphopoiesis [30, 31]. As the number of B cell precursors normally decreases in tumor bearing mice [32, 33], it must be speculated that in our experiment of bone marrow suppression, c-Kit blockade stimulates B cell genesis and thus increases tumor cell engraftment. Furthermore, B lymphopoiesis is associated with increased angiogenesis and cellular proliferation [34–36]. In our study, neoangiogenesis within the colorectal tumors of animals pretreated with anti-c-Kit was comparable to controls despite the lack of functional HCs which are necessary for angiogenesis and vasculogenesis. Thus, we hypothesize that the lack of HCs was compensated by local angiogenic factors or B lymphopoiesis-associated angiogenesis, resulting in a tumor neovascularization comparable to that observed in controls.

The SCF receptor c-Kit has been shown to be essential for the development of blood cells, melanocytes, germ cells, interstitial cells of Cajal in the gastrointestinal tract, and mast cells [37]. Furthermore, the majority of tumor cells, especially those of the neural axis, breast, lung, prostate, and colon show an aberrant c-Kit expression [38]. Especially gastrointestinal stromal tumors (GISTs) express c-Kit on the cell-surface, and mutations of Kit in these tumors results in an activation of Kit signaling, which leads to uncontrolled cell proliferation and resistance to apoptosis [39, 40]. Today, patients with GIST are treated with Imatinib, an inhibitor of certain protein tyrosine kinases including KIT, depending on the mitotic index of the GIST. Imatinib induces an arrest of tumor cell proliferation and causes apoptotic cell death. In established MCA26 tumors, Pan et al. showed that injection of anti-c-Kit antibodies markedly reduces tumor-induced immune tolerance exhibited by myeloid-derived suppressors in mice [41]. Furthermore, their experiments demonstrate that anti-c-Kit treatment can prevent tumor-specific T-cell anergy and development of T regulatory cells (Treg). In combination with an immune modulatory therapy of IL-12 plus 4-1BB activation, treatment with anti-c-Kit antibodies significantly improved the long-term survival of MCA26 tumor bearing mice [41]. In our study, only 8% of the CT26.WT-GFP cells were c-Kit receptor positive. Therefore, we do not expect a direct inhibitory effect of anti-c-Kit treatment on tumor cell proliferation. 

Recruitment of HCs and EPC is predominantly mediated by SDF-1 and its receptor CXCR4 [9, 10] because HCs and EPCs migrate along a chemotactic gradient towards higher concentrations of SDF-1 [12–14]. Kaminski et al. have shown that almost 100% of bone marrow and peripheral blood c-Kit^+^ cells are positive for CXCR4 [12]. In the present study, we could demonstrate that neutralization of SDF-1 after anti-c-Kit pretreatment significantly reduces neovascularization most probably by the inhibition of HC and EPC recruitment within the tumors. Additionally, in combination with anti-c-Kit pretreatment, SDF-1 neutralization is capable of decreasing tumor cell proliferation and invasion. Taken together, neutralization of SDF-1 counteracts the stimulating effects of bone marrow suppression by anti-c-Kit treatment on tumor cell engraftment and inhibits compensatory local and B lymphopoiesis-associated angiogenesis.

As described by Kaminski et al., the presence of SDF-1 chemoattractant activity and inflammatory endothelial activation by TNF-*α* is required for c-Kit^+^ cells to form functionally relevant interactions with the endothelium in postcapillary venules [12]. Blockade of ICAM-1 and CXCR4 abolishes adhesion of c-Kit^+^ cells to the vascular endothelium despite application of SDF-1 and TNF-*α*. Moreover, in their cremaster muscle microcirculation model, stem cell adhesion was significantly reduced when eNOS was not present or systemic NOS inhibited [12]. As SDF-1 and TNF-*α* stimulation activates the endothelium by an increase of the CXCR4 expression leading to relevant stem cell attraction [12], our results indicate that the increase of CXCR4 and VEGF expression within the tumors represents a compensatory pathway after anti-c-Kit pretreatment. Neutralization of SDF-1 inhibits interactions of c-Kit positive cells with tumor vessels and as a consequence, leads to inhibition of tumor neovascularization.

In conclusion, bone marrow suppression by anti-c-Kit pretreatment significantly enhances tumor cell engraftment of colorectal tumors. As anti-SDF-1 treatment counteracts this increased tumor outgrowth by inhibition of neovascularization, the SDF-1/CXCR4 pathway seems to be crucial for tumor angiogenesis mediated by HCs and EPCs.

## Figures and Tables

**Table 1:** Microvessel diameters within the tumor margin and the tumor center of control animals, animals pretreated with anti-c-Kit and animals pretreated with anti-c-Kit followed by anti-SDF-1 treatment. All values are given in *μ*m. Diameters slightly increased during the observation period. No significant differences could be observed between the tumor margin and center. Fourteen days after tumor cell implantation, microvascular diameters within the tumor center were significantly smaller in anti-c-Kit/anti-SDF-1 treated animals compared to controls and to anti-c-Kit treated animals.

	Time	Control	Anti-c-Kit	Anti-c-Kit/anti-SDF-1
Tumor margin	d5	12.42 ± 0.53	11.88 ± 0.77	13.02 ± 0.52
	d8	13.97 ± 0.48	14.60 ± 0.93	15.02 ± 0.57
	d11	14.73 ± 1.36	15.39 ± 1.00	14.84 ± 1.06
	d14	15.81 ± 1.20	15.24 ± 0.91	13.94 ± 0.75

Tumor centre	d5	13.91 ± 0.67	12.85 ± 0.83	13.22 ± 1.00
	d8	14.44 ± 0.52	13.93 ± 0.75	14.21 ± 0.47
	d11	15.35 ± 1.40	15.26 ± 0.98	15.66 ± 1.83
	d14	16.86 ± 1.10	16.84 ± 1.35	12.82 ± 0.67^∗#^

Data are given as mean ± SEM; *P < 0.05 versus control; ^#^ *P* < 0.05 versus anti-c-Kit.

**Table 2:** Tumor cell migration in dorsal skinfold chambers of control animals, animals pretreated with anti-c-Kit, and animals pretreated with anti-c-Kit followed by anti-SDF-1 treatment during a 14-day observation period. Migrated tumor cells next to the tumor margin were observed from day 5 until the end of the observation time. Comparing all three groups, no significant differences could be observed concerning the total number of migrated cells as well as their distance [*μ*m] from the tumor margin. Of interest, at the later time points, tumor cells were found at greater distances from the tumor margin.

	Time	Control	Anti-c-Kit	Anti-c-Kit/anti-SDF-1
Number	d5	22.08 ± 1.33	23.00 ± 1.33	19.91 ± 1.14
	d8	22.67 ± 1.33	23.14 ± 1.18	20.67 ± 1.07
	d11	22.42 ± 1.53	23.48 ± 1.05	20.80 ± 1.34
	d14	20.84 ± 1.71	23.96 ± 1.32	21.17 ± 1.30

Distance	d5	276.24 ± 16.08	291.82 ± 15.95	309.45 ± 50.93
	d8	337.94 ± 9.38	351.11 ± 23.57	323.38 ± 20.45
	d11	383.85 ± 15.80	423.32 ± 25.65	360.83 ± 18.58
	d14	421.50 ± 22.40	438.49 ± 32.01	458.51 ± 59.01

Data are given as mean ± SEM.
